# Universal parental support-How to reach out: a cross-sectional random sample of Swedish parents

**DOI:** 10.1186/1471-2458-14-1064

**Published:** 2014-10-11

**Authors:** Karin Thorslund, Jan Johansson Hanse, Ulf Axberg

**Affiliations:** Department of Psychology, University of Gothenburg, Gothenburg, Sweden; Nordic School of Public Health NHV, Gothenburg, Sweden

**Keywords:** Universal prevention, Parent training, Parental engagement, Adolescence, Parental support

## Abstract

**Background:**

Young children in Sweden have good general health in comparison to children in other European countries. In contrast, teenagers display poorer mental health. Parental support is now being made available on a universal level in Sweden in order to promote youngsters general psychological health. The aim of this study was to examine (1) to what extent the parents were interested in various forms of municipality-based parental support programs; (2) whether there were any differences between mothers and fathers as regards their interest in municipality-based parental support programs; and (3) if there were any differences between high to non-users of the Internet as an information source in their parenting, regarding their interest in municipality-based parental support programs.

**Methods:**

The study was based on a random sample of parents in 15 municipalities in Sweden. Telephone interviews were conducted with 1744 parents. The information collected included the parent’s gender, number of children, age of children, what municipality-based support parents would be interested in, and information about the use of the Internet as an information source in their parenting.

**Results:**

The results showed that there was a significant difference between mothers and fathers regarding interest in parental support, with mothers being more interested in all forms of parental support except a webpage for parents. Additionally, the results show that high frequent use of the Internet as an information source in their parenting was associated with high interest in municipality-based parental support.

**Conclusions:**

Parents who are active in seeking web-based information about their child and parenting are also parents interested in various kinds of parental support. The municipality is generally better at evoking the interest of mothers than fathers concerning all forms of support, except a webpage with information for parents. Municipalities should develop attractive and informative webpages for parents, with some information specifically addressing fathers.

## Background

The mental health of children and adolescents is a priority area in Sweden [1]. From an international perspective, younger children in Sweden have comparably good psychological health [1]. When asked about their general health, Swedish children in 5th grade describe themselves as equally healthy or healthier than children did 20 years ago, which is well in line with the general development of public health in Sweden [2]. However, youths in 9th grade describe themselves as less healthy. This is alarming, and it seems to be to be a specific Swedish problem since the development contradicts the development in the other Nordic countries and the rest of Europe, which seems to go in a more positive direction [3, 4]. The Swedish government has expressed its concern about this development and conducted an inquiry, which concluded that probably the most influential protective factors in a child’s life are the family and the parenting capacity of the parents [2, 5]. The inquiry emphasizes the complexity of parenting in modern society and stresses the need for provision of universal parental support of various kinds [5].

Parenting style is among the parameters that have sparked the most interest in parenting research [6]. Ever since Baumrinds’ [7] research on parenting control and warmth in the 1960s, different parenting practices have been demonstrated that either increase or decrease the risk of negative developments in children’s psychological health. Whereas psychological control (e.g. through love withdrawal, induction of guilt, invalidation of feelings, restriction of verbal expression, etc.) has been linked to increased risk of internalizing problems especially among girls, behavioral control (through limit setting, age-appropriate demands, monitoring, etc.) has been linked to increased well-being in children [8]. Lack of warmth, poor parental responsiveness to the child, harsh and/or inconsistent limit-setting, poor monitoring, inappropriate developmental expectations and parental reinforcement of disruptive behavior are examples of parenting practices that have been shown to increase the risk of a negative development [9–14]. Authoritative parenting, parental sensitivity, warmth structuring and non-hostility, as well as parents satisfaction with social and emotional support have been connected with positive developments and may serve as a protective factor [15]. Parental cognitive factors, such as self-efficacy and attitudes in relation to the child, have been found to be important factors in parenting [16].

During the last decade, several parenting training programs aimed at promoting positive and preventing negative developments have been introduced and spread in Sweden [17–19]. Although they differ in which types of parents they target, with regard to influences from different theories, group size, number of sessions, and the themes each method focuses on most, a common goal for the different programs is to promote positive and reduce harsh and inconsistent parenting. Several of these programs have been recommended for universal use by the Swedish Institute of National Health [20]. While most of the programs still need to be evaluated for effect when used in a universal context, a shortened version of the program IYS (Incredible Years Series has been evaluated in Norway. It was found to reduce harsh parenting (moderate to large effects) and children’s’ behavior problems (small effects), and enhance positive parenting (large effects), and parents’ sense of competence (small effects) [21].

There is thus a large body of available research on parenting. However, the research has mainly been focused on mothers and mothers’ parenting behaviors and not taken fathers’ parenting or systemic aspects into account [22]. Even if the father’s active and regular engagement in the child has been shown to be predictive of positive behavioral and psychological outcomes in the child [23], and even if many fathers want to be involved with their children, little or no help at all is offered specifically to the majority of these men regarding parenting [24]. Thus, arguments are raised to urge both professionals and policy makers to improve circumstances for increased involvement of fathers in their children’s care and development [23–26]. Fathers increased involvement in family life is much needed. Since 1999, 80 percent of Swedish women are in the workforce [27]. However, women still take the lion’s share of the parental leave, which leads to inequality at work and at home [28]. The gender differences in sick leave have increased during the last 30 years [29]. The main difference consists of womens’ sick leave increasing after the first child, and increasing even more after the second child [29].

A crucial issue when aiming support universally is how to reach out, and how to reach out in a way that will include fathers from the start. In a systematic review, the authors conclude that parents use the Internet as a source of information, and for social support, but also that the Internet contains an abundance of information that can be either useful or contradictory and misleading [30]. In Sweden, people in general have access to, and use, the Internet. Sweden is ranked number one in the world on the World Wide Web Index, with the best web infrastructure, best web usage (percentage of web users and content available), and the highest impact of the web on social, economic and political dimensions [31].

The phenomenon when use of the Internet as a resource for information is unequally distributed in different socio-economic groups is known as the “digital divide” [32]. In Sweden, however, when studying users of a large parenting website, usage was found not to follow the “digital divide,” but instead to reveal a surprising lack of fathers. This would indicate that the Internet as a resource for parents in Sweden may be a socially unbiased, but instead gender biased, medium [33]. On the other hand, the parenting website in question features only female writers, and a compelling majority of the presented experts are women, and so as a visitor one might perceive the site to be aimed more towards mothers than fathers. When both parents feel equally addressed, the interest among fathers may be greater. This is supported by another Swedish study, where the effect of an online parent training program (Comet) was measured. When the program was offered online, 69% of the parents participated together, instead of the usual 8% when the same program is offered to groups of parents in real life, the other participants in such cases being only mothers [34].

In order to meet the challenges of parenting in the modern society, the Swedish government has formulated a national strategy for parenting support [5]. The strategy is a broad approach with many different kinds of initiatives from parent education in antenatal clinics during pregnancy to the provision of various initiatives on the universal level, and addresses all parents of children between the ages 0 and 17 years, i.e. all parents are offered the same opportunities for support and help. The national strategy proposes that parental support should include activities that aim to give parents knowledge of children’s health and children’s emotional, cognitive and social development as well as to enhance parents’ social networks. Priority areas thus include universal parental support programs that aim to offer parents support and help in their parenting, individual counseling for parents by trained counselors, places where parents can meet and connect with other parents, and the provision of parenting support by municipal telephone or Internet services [2].

In Sweden, the municipalities already offer universal support to some extent through antenatal clinics, childcare, schools, the churches and volunteer organizations. In each municipality, support is offered in a range of different forms. Before developing universal support to parents, one should consider what they are most interested in benefitting from, and how to reach out to both mothers and fathers. Since mothers take on more responsibility at home [28, 29], we expected mothers to show greater interest in all forms of parental support.

### Aim

The aim of the current study was to examine (1) to what extent the parents were interested in various forms of municipality-based parental support programs; (2) whether there were any differences between mothers and fathers as regards their interest in municipality-based parental support programs; and (3) if there were any differences between high to non-users of the Internet (as an information source in their parenting) regarding their interest in municipality-based parental support programs.

## Methods

### Procedure

Fifteen municipalities implemented a project supported by the National Institute of Public Health in Sweden, along with a research team at the Department of Psychology, University of Gothenburg. The project was called “Development of municipality based strategies for parental support” and was commissioned to the National Institute of Public Health by the Swedish government. The aim of the project was to develop and extend the universal parental support in each municipality. The timeline for the project was from January 2010 to December 2011. The first step, before adding and implementing new forms of parental support, was to determine what forms of support was already available in each municipality, and parents’ perceived need and requests for support. As a part of this process, a telephone survey was performed in order to find out what parents living in the area would be interested in, in terms of parental support. The research team has closely followed the work of the municipality in mapping parents interests in, and requirements of, support.

Before starting the interview the interviewer would introduce themselves, explain where they were calling from and the purpose of the interview, and ask for informed consent by saying: I would like to ask you permission to ask you a few questions regarding this. It would take a few minutes and your answers will be coded so that it will not appear who answered the questions. The interviewer would use the home telephone number, but would interview either the mother or the father, depending on who answered the phone first. When the interview was finished, the interviewer would ask permission to interview the other parent. If the mother was not living with the father, the interviewer would ask for his number and call him. If the other parent was not at home, the interviewer would ask permission to call back and ask when this would be most convenient.

### Participants

A random sample of parents, and their contact info, was acquired from the Swedish Population and Address Registry (SPAR). The registry provided the contact info of the parent listed as the primary caregiver of children 0–17 years old. Normal procedure for this registry is to list the mother as primary caregiver. Hence, the list contained phone numbers of mainly mothers. Fathers had to be contacted through the mother’s phone number in most cases. A total of 2136 parents were contacted by phone (answered the phone call) and 1744 of them agreed to participate in the interviews (response rate: 82 percent). The response rate for mothers was 83 percent and the response rate for fathers was 81 percent. The proportion of mothers was 65 percent and the proportion of fathers was 35 percent. Twenty-six percent of the participants had one or more children who were 1–36 months, 72 percent had one or more children aged 3–12 years, and 38 percent had one or more teenagers.

### Measures

A semi-structured interview guide was developed, in cooperation with a research group at Örebro University who had performed a telephone interview for similar purposes, and in cooperation with the public health coordinators in the municipalities. Background questions included number of, and age of, children and parents gender. The interview questions were “*If you were invited to sign up for courses or have available advice and support to learn more about how you as a parent can create good conditions for your child, do you think you would take advantage of that opportunity in the form of a*:” followed by the alternatives “*leader-led parent training program*” (yes/no/maybe); “*a meeting room where you could meet other parents*” (yes/no/maybe); “*individual counseling*” (yes/no/maybe); “*a municipality-based phone line for parents*” (yes/no/maybe); “*a municipality-based webpage for parents*” (yes/no/maybe). “*Do you use the Internet as a source of information for your parenting?*” (No, never/Daily/Around once a week/Around once a month/More seldom)

### Ethics

We have followed the Swedish law, “Ethical Review of Research involving Humans” [35, 36]. In accordance with the law, ethical approval was not required since sensitive personal data was not collected. The interviewees took part of information that followed the WHO ethical recommendation [37]. A research application was submitted to the Swedish National Institute of Public Health (registration number HFÅ 2009/215). The review board at the Swedish National Institute of Public Health has approved the project without any requirements on further ethical vetting.

### Statistical analysis

The chi-square test was used to compare proportions, e.g. between maternal and paternal responses. Standardized residuals were used to examine which cell(s) were major contributors (an absolute value greater than 2) to the significant chi-square value [38]. Oneway analysis of variance (ANOVA) with Scheffe’s post hoc tests were used to test differences between means. The level of significance was set at p < .05. Statistical analyses were performed using SPSS Statistics version 20.

## Results

One of the questions in the interview concerned five hypothetical offers of parental education and support to create good conditions for their child, based on the areas prioritized by the Swedish government. The question was whether or not the parent would attend/use these options. Five examples of municipality-based education or support were given in the interview and Table 1 shows the proportions of interest. The greatest interest was for a webpage for parents (65.7 percent overall for mothers and fathers). As expected, the proportions of interest were significantly different between mothers and fathers as regards: leader-led, Chi-square (2, N = 1713) = 30.36, p < .001, meeting room, Chi-square (2, N = 1713) = 20.81, p < .001, individual counseling, Chi-square (2, N = 1717) =22.69, p < .001, municipality-based phone, Chi-square (2, N = 1714) =24.41, p < .001. For these four variables, the fathers answered ‘no’ in a significantly higher degree (standardized residuals were between 2.8 to 3.4). Interestingly, the variable concerning interest in a webpage for parents did not show a significant difference between mothers and fathers, Chi-square (2, N = 1716) =5.24, p = .07.

**Table 1:** **Parental interest and gender**

Type of municipality-based parental support	Interest in municipality-based parental support	Parents’ gender	
		Mother (N = 1123-1126) ^1^	Father (N = 586-589) ^1^
Leader-led	Yes	49	36
	Maybe	23	24
	No	28	40
Meeting room (opportunities to meet other parents)	Yes	48	38
	Maybe	17	17
	No	35	45
Individual counseling	Yes	56	44
	Maybe	19	21
	No	25	35
Parenting phone line	Yes	49	37
	Maybe	16	17
	No	35	46
Webpage for parents	Yes	67	63
	Maybe	14	14
	No	19	23

^1^N varies for the different forms of parental support due to missing answers.

Interest in various forms of municipality-based parental support, broken down by parents’ gender (percent, rounded figures).

Parents were also asked about their actual use of the Internet as an information source in their parenting. A majority of the parents used the Internet for parenting information either monthly/rarely (51 percent) or daily/weekly (9 percent). However, 40 percent of the parents answered that they never use the Internet as an information source in their parenting. This question about the parents’ use of the Internet as an information source in their parenting was cross-tabulated with the question on whether or not the parents would attend/use various options about municipality-based parental support programs to create good conditions for their children. Table 2 shows that the proportions of interest in municipality-based education and support were significantly different between high and non-users of the Internet as an information source in their parenting: leader-led support, Chi-square (4, N = 1713) =77.76, p < .001, meeting room, Chi-square (4, N = 1714) =69.72, p < .001, individual counseling, Chi-square (4, N = 1717) =31.27, p < .001, municipality-based parent phone, Chi-square(4, N = 1714) =35.01, p < .001, interest in a webpage for parents, Chi-square (4, N = 1716) =97.58, p < .001. Non users of the Internet as an information source in their parenting answered ‘no’ at a significantly higher degree to all five questions about municipality-based parental support (standardized residuals were between 2.7 to 6.2). For example, the interest in a webpage for parents was 85 percent among the high-frequent Internet users, and 55 percent among the non-users of the Internet (as an information source in parenting). In Table 2, a dose response relationship seems apparent for most variables, interest in each type of support increasing as internet usage goes from never to monthly to daily.

**Table 2:** **Parental interest and use of internet**

Type of municipality-based parental support	Interest in municipality-based parental support	How often parents’ use the internet as an information source in their parenting		
		Never (N = 684)	Monthly, rarely (N = 874)	Daily, weekly (N = 148)
Leader-led	Yes	34	51	54
	Maybe	22	25	23
	No	44	24	23
Meeting room (opportunities to meet other parents)	Yes	35	50	60
	Maybe	16	19	13
	No	49	31	27
Individual counseling	Yes	50	51	64
	Maybe	16	24	17
	No	34	25	19
Parenting phone line	Yes	41	44	60
	Maybe	13	20	13
	No	46	36	27
Webpage for parents	Yes	55	71	85
	Maybe	14	15	5
	No	31	14	10

Interest in various forms of municipality-based parental support, broken down by the parents’ use of the Internet as an information source in their parenting (percent, rounded figures).

In total, the parents answered five questions about whether or not they would attend/use the option about municipality-based parental education and support to create good conditions for their child. These five questions were categorized as follows: parents who answered “yes” to all five questions were given a specific code number, i.e. these parents answered that they have interest in participating in all five municipality-based parental support programs. The other combinations of answers were given a different code number, the same for all combinations. Descriptive statistics showed that 12.7 percent (N = 219) were interested in all five forms of municipality-based parental support. These categorized answers about parents’ interest in attending/using the option about municipality-based parental support programs was then cross-tabulated with parents’ use of the Internet as an information source in parenting, see Table 3.

**Table 3:** **Interest and internet**

Interest in municipality-based parental support programs	How often parents use the Internet as an information source in their parenting		
	Never (N = 684)	Monthly, rarely (N = 874)	Daily, weekly (N = 148)
Yes, interest in all five	10	14	20
Not interested in all five (including no interest at all)	90	86	80

Interest in all five municipality-based parental support programs, broken down by the parents’ use of the Internet as an information source in their parenting (percent, rounded figures).

Overall, the results showed significant differences between different combinations of categories, Chi-square (2, N = 1706) =12.00, p < .01. The cell that contributed most to the overall result was the combination of daily/weekly use of the Internet as an information source in parenting and interest in all five municipality-based parental support programs, with a standardized residual of 2.3, which means that parents who use the Internet daily/weekly also to a greater extent answered that they are interested in all five municipality-based parental support programs. As can been seen in Table 3, 20 percent of the daily/weekly users of the Internet were interested in all five parental support programs, whereas among the non- users of the Internet (as an information source in parenting), only 10 percent were interested in all five parental support programs.

The differences between the groups of internet users regarding levels of interest in municipality-based parental support programs were also explored using one way analysis of variance (ANOVA). The parents’ use of the Internet as an information source in parenting was categorized into the groups “Never”, “Once a month/more seldom”, and “Daily/Once a week”. The dependent variable was number of forms of parental support the parent would say “Yes” to (0–5). The group who had answered that they used the Internet as an information source in their parenting daily or once a week also answered “Yes” to more forms of parental support (*M* =3.22) compared to parents who reported using the Internet for the same purpose once a month/more seldom (*M* =2,66) or never (*M* =2.15). The differences in interest in the various forms of support between the three groups were significant, *F*(2, 1714) =3656, *p* < .001, see Table 4.

**Table 4:** **Interest and internet**

Interest in municipality-based parental support programs	How often parents use the Internet as an information source in their parenting			F (2,1714)
	Never (N = 684)	Monthly, rarely (N = 874)	Daily, weekly (N = 148)	
“Yes”	2,15 (1,65)*	2,66 (1,55)*	3,22 (1,37)*	3656

*p < .001 according to ANOVA and Sheffe’s post hoc tests.

Mean of interest in all five municipality-based parental support programs, and the parents’ use of the Internet as an information source in their parenting.

## Discussion

The results showed that there was an expected significant difference between mothers and fathers regarding interest in parental support, with mothers being more interested in all forms of parental support, except a webpage for parents, which is an unexpected finding. Additionally, the results show that high frequent use of the Internet as an information source in their parenting was associated with high interest in municipality-based parental support. Frequency of internet usage and interest in various forms of parental support seems to have a dose response relationship.

One interpretation of the results from the current study (see Table 2) is that if you are active in using the Internet for parenting information it may be viewed as an indicator of increased interest in other forms of parental support also. Less active information seeking on the Internet seems to demonstrate a correlation with being clearly less interested in getting help and advice, no matter what kind of municipality-based support you are offered. Additionally, these results were supported when comparing the group of parents who expressed interest in all five parenting support programs, as these parents also were very active in using the Internet for parenting information. Thus, these results indicate that there are interesting challenges in trying to reach the group of parents who do not actively seek parenting information. The results also indicate that the Internet is a good channel of information to reach out to parents who are interested in parental support. In general, we believe that the Internet is an important resource in parenting, particularly to acquire knowledge about parenting and child health. Another conclusion is that there is a very strong interest in a municipality-based website with information about parenting and child health. More than half of the parents who have never looked up information about parenting on the Internet claim to be interested in a local webpage for parents. This supports the conclusion that a webpage can be a powerful tool in reaching out to parents, that most parents in Sweden today are comfortable with searching on the Internet, and that for many people the Internet is the first source of information. This strategy would, however, pose demands on developing webpages that are attractive to the public and of good quality, since they would compete with existing commercial pages aimed towards parents.

Other possible arenas for spreading information about the municipality-based webpage, and for offering parental support of various forms are, for example, through the existing childcare and school systems, and through the primary care system. All parents pass through these systems, there is research indicating that these are the arenas preferred by parents for parental support [39] and the professionals in these systems have adequate competencies. Advice and support is already being offered in primary child health care, so adding more parental support would fit in this context. School meetings are also a context where universal parental support would fit in easily. Other important arenas are the non-governmental organizations where children go for activities. Using all these arenas could be a way of also reaching the parents who do not actively seek information or support.

A possible conclusion is that the generally lower interest among fathers in parental support may not only reflect fathers’ lower involvement in parenting, but perhaps also the failure of the existing municipality-based services in addressing fathers in contacts with the family regarding the child. The current study found equal interest in municipality-based support via a webpage. This is an interesting finding. It may reflect the fact that this is a new arena for encounters between the municipality and the public, gender patterns have not yet been established, and everything is still possible. When introducing webpages for parents, it is of great importance that all parents feel equally addressed, regardless of gender.

One way to further enhance the active involvement of fathers may be to adapt the manner of providing information about universal parent support programs. The current study shows that municipality-based online information (webpage for parents) can be an important and specific way to reach fathers in a better way. In general, this is also supported by the Swedish government’s national strategy for parenting support, where one of the approaches is to utilize the Internet in order to get information out to the parents [2]. However, the national strategy does not specifically address the importance of the Internet for the fathers. Another way is to offer parental training as online courses, since it has been found that fathers tend to participate in online courses along with the mother to a greater extent (69 percent of fathers participated) compared to group settings (8 percent of fathers participated) [34].

### Strengths and limitations

A number of limitations need to be considered regarding the current study. The measures used in this study are limited. The interview guide was developed in cooperation with other researchers and tailored to the involved municipalities interests of knowledge, and has not been published or researched for validity. Furthermore, we do not have information about child behavior and any emotional problems. Heinrichs et al. [40] found that parents of children with higher levels of problems were more likely to participate in a universal prevention program. In the current study, it was not possible to analyze the occurrence of child problems and parents’ interest in and knowledge about universal parent support programs. Neither do we have information about parents SES (Socioeconomic Status), and cannot relate the levels of interest to this variable. Another limitation is that the proportion of mothers is bigger than the proportion of fathers. The registry used for contact info routinely lists the mother as the contact person of the child, so the lists of contact info contained mostly mothers. The mothers did not always live with the father, and in these cases the phone number of the father had to be obtained through the mother (or vice versa, in some cases). Also, mothers answered the phone, and were at home, more frequently, and thus would be interviewed more frequently. However, when fathers answered the phone, they would agree to participate in the interview to the same extent as mothers. The current study has several strengths. The study is based on a big random sample (n = 1744) with a high response rate (82 percent). This means that although the proportion of fathers is smaller than that of mothers, the number of fathers is still substantial.

### Recommendations for future research

Future research on universal parent support programs might investigate the group of parents who decline interest in any parental support, and who do not seek information regarding parenting on the Internet. Is it a homogeneous group or a heterogeneous group, and is there a true need for support or not? If there is a need for parental support, how could one tailor parental support to fit this group, if universal parental support is not an alternative? It is of great interest to try to understand why some parents are not interested in parental support. Is this because municipalities offer inadequate support, at the wrong time? Or could it be other factors that are related to the family situation? In order to gain an in-depth understanding of barriers, thoroughly conducted qualitative studies would be required. It would also be interesting to be able to relate parents interest in universal support to SES, since research shows that economically disadvantaged families and families with children with more severe behavior problems are more likely to enroll in and complete community-based than clinic-based parent training programs [41].

## Conclusions

The current study and previous studies support recommendations of some municipal strategies regarding parenting [37]. The Internet is an important but challenging information channel to reach out to parents today with information regarding parenting and child health. Internet use in seeking for hints and support in parenting seems to be a firm indicator of interest in other forms of parental support. Parents who are active in seeking information about their child and parenting are also parents interested in various kinds of parental support. In contrast, parents who never use the Internet in search of advice and information seem not to be interested in other forms of parental support.

The strong correlation between frequency of Internet use and interest in parental support confirms the notion that the Internet is a good way to reach those who are interested in parental support. However, to reach the group who would benefit from parental support but is not interested is a challenge, and too much hope should not be put in the Internet, but rather in other innovative ways such as other forms of media, as a conversation topic from parent to parent, or in other arenas where parents meet such as children’s schools.

The municipality is generally better at evoking the interest of mothers than fathers concerning all forms of support, except as regards webpages with information for parents, where interest is relatively equal for both parents.

In addition, municipalities should consider various municipal alternatives for parenting support programs. One way to reach a larger group of parents can be to offer distance learning using web-based courses in parenting.
